# Estimation of External Dose by Car-Borne Survey in Kerala, India

**DOI:** 10.1371/journal.pone.0124433

**Published:** 2015-04-17

**Authors:** Masahiro Hosoda, Shinji Tokonami, Yasutaka Omori, Sarata Kumar Sahoo, Suminori Akiba, Atsuyuki Sorimachi, Tetsuo Ishikawa, Raghu Ram Nair, Padmavathy Amma Jayalekshmi, Paul Sebastian, Kazuki Iwaoka, Naofumi Akata, Hiromi Kudo

**Affiliations:** 1 Hirosaki University Graduate School of Health Sciences, Aomori, Japan; 2 Institute of Radiation Emergency Medicine, Hirosaki University, Aomori, Japan; 3 Fukushima Medical University, Fukushima, Japan; 4 National Institute of Radiological Sciences, Chiba, Japan; 5 Kagoshima University Graduate School of Medical and Dental Sciences, Kagoshima, Japan; 6 Regional Cancer Center, Kerala, India; 7 Natural Background Radiation Cancer Registry, Kerala, India; 8 National Institute for Fusion Science, Gifu, Japan; University of South Carolina, UNITED STATES

## Abstract

A car-borne survey was carried out in Kerala, India to estimate external dose. Measurements were made with a 3-in × 3-in NaI(Tl) scintillation spectrometer from September 23 to 27, 2013. The routes were selected from 12 Panchayats in Karunagappally Taluk which were classified into high level, mid-level and low level high background radiation (HBR) areas. A heterogeneous distribution of air kerma rates was seen in the dose rate distribution map. The maximum air kerma rate, 2.1 μGy/h, was observed on a beach sand surface. ^232^Th activity concentration for the beach sand was higher than that for soil and grass surfaces, and the range of activity concentration was estimated to be 0.7–2.3 kBq/kg. The contribution of ^232^Th to air kerma rate was over 70% at the measurement points with values larger than 0.34 μGy/h. The maximum value of the annual effective dose in Karunagappally Taluk was observed around coastal areas, and it was estimated to be 13 mSv/y. More than 30% of all the annual effective doses obtained in this survey exceeded 1 mSv/y.

## Introduction

Large amounts of artificial radionuclides were released from the reactor buildings into the environment by the 2011 Fukushima Dai-ichi Nuclear Power Plant accident [1]. External and internal dose estimations were carried out by government and university researchers immediately after the accident [2–4]. In a 2013 report, the United Nations Scientific Committee on the Effects of Atomic Radiation (UNSCEAR) reported that no apparent increase of cancer incidence due to this accident is expected in the future [5]. However, the biological effects due to radiation exposure have become a large concern among Japanese residents. Recently, a review paper was published on the effects of natural variation in natural radionuclides on humans, animals and other organisms [6]. Since the available studies are few in number however, it is difficult to conclude what the effect is of chronic exposure by low level radiation. The authors proposed a three-year project (from July 2012 through March 2015) to look at the health risk associated with exposure to low-dose-rate ionizing radiation. The high background radiation (HBR) area of Kerala state was selected from which prediction data could be obtained for the project. Kerala state is located in southern India and is well-documented as a high background radiation area [7]. The annual absorbed dose for the coastal areas of Karunagappally Taluk has been observed to have especially high values due to thorium-containing monazite sand found there and external dose estimation was carried out for this area as a part of an epidemiological study [8]. However, it is necessary to make a dose distribution map for a detailed evaluation of external dose and no such map has been made yet for this area. Thus, a car-borne survey was carried out in Karunagappally Taluk for the purposes of making a dose distribution map and estimating the annual external dose there.

## Materials and Methods

### Ethics Statement

No specific permissions were required for these locations/activities for the following reasons; a) The field studies did not involve endangered or protected species; b) We provide the specific locations of our study (GPS coordinates).

### Survey area

**Fig 1:**
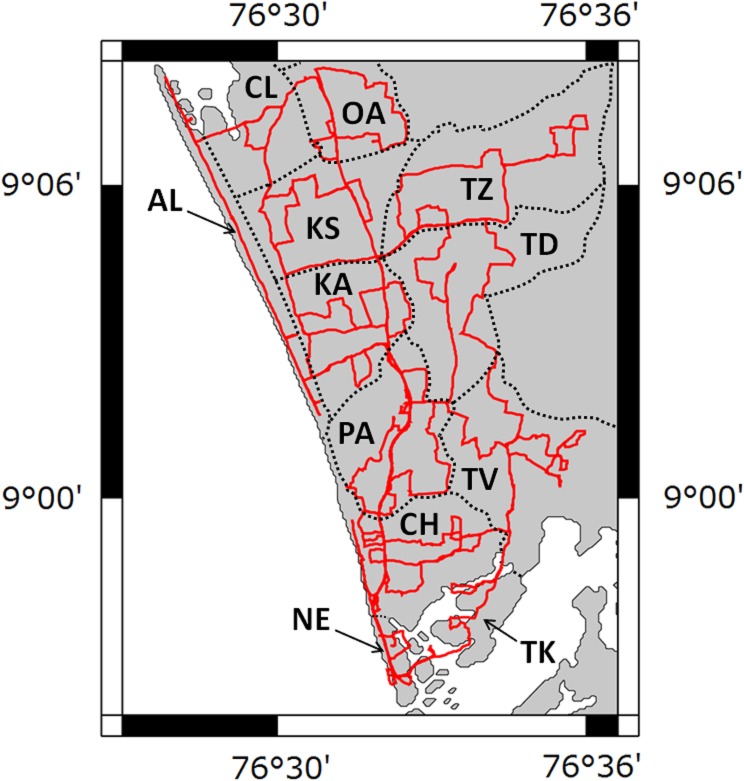
The route map in Karunagappally Taluk. The abbreviations of each Panchayat are shown in the map and are as follows. AL: Alappad, CH: Chavara, CL: Clappana, KA: Karunagappally, KS: K. S. Puram, NE: Neendakara, OA: Oachira, PA: Panmana, TD: Thodiyoor, TK: Thekkumbhagam, TV: Thevalakkara, TZ: Thazhava.

Karunagappally Taluk, in Kerala state, India consists of 12 Panchayats which are administrative sub units [8]. The survey route which covered all the Panchayats of Karunagappally Taluk is shown in Fig 1. This route map was drawn using the Generic Mapping Tools (GMT) created by Wessel and Smith [9]. The 12 Panchayats were classified as low level, mid-level, and high level HBR areas according to the annual absorbed doses. Oachira, Thazhava, Thevalakkara, and Thodiyoor Panchayats were low level HBR areas; Clappana, Karunagappally, K. S. Puram, and Thekkumbhagam Panchayats were mid-level HBR areas; and Alappad, Chavara, Neendakara, and Panmana Panchayats were high level HBR areas.

### Car-borne survey

A car-born survey technique is a convenient method for the evaluation of radiation dose in a wide area in a short period [10]. Thus, a car-borne survey which used a 3-in × 3-in NaI(Tl) scintillation spectrometer (EMF-211, EMF Japan Co., Japan) was carried out in Karunagappally Taluk from September 23 to 27 in 2013. Measurements of the counts inside the car were carried out every 30 s along the route. Simultaneously with the gamma-ray count measurements, the latitude and longitude at each measurement point were measured with a global positioning system. Car speed was kept around 40 km/h. Since count rate was measured inside the car, it was corrected by multiplying with a shielding factor in order to represent the unshielded external dose rate. The shielding factor of the car body was estimated by making measurements inside and outside the car at 34 points and correcting them with the inside count rates.

In this study, a response matrix method was used to convert the gamma-ray pulse height distribution measured with a NaI(Tl) scintillation spectrometer to the energy spectrum of incident gamma-rays [11, 12]. The details of the response matrix method are described as follows. The photon peaks of ^40^K (*E*_γ_ = 1.464 MeV) and ^208^Tl (*E*_γ_ = 2.615 MeV) were used for calibration from the channel number to gamma-ray energy. The photon peak positions of ^40^K and ^208^Tl were determined accurately by smoothing the gamma-ray pulse height distribution in Fourier expansion [13].

Since it is difficult to obtain the photon peak for each gamma-ray in a 30-s measurement in the natural environment, the accuracy of the energy calibration will be low. Furthermore, photon peaks obtained with the NaI(Tl) scintillation detector are affected by temperature, and photon peak positions of gamma-rays from ^40^K and ^208^Tl will drift from side to side [13]. Thus, accuracy of the air kerma rate which is estimated using a gamma-ray pulse height distribution obtained by 30-s measurements will be low. Accordingly, the relationship between the total counts of a gamma-ray pulse height distribution and air kerma rates was examined for the estimation of dose rate conversion factor. Measurements of gamma-ray pulse height distribution were carried out 1 m above the ground surface at 35 points. At one measurement site, the gamma-ray pulse height distribution was obtained at two different points. So the number of measurements for the estimation of dose rate conversion factor differed from the number of measurements for the estimation of shielding factor. Counting times at high level areas, mid-level areas and low level areas were set as 300 s, 600 s and 900 s, respectively. The weather condition was sunny throughout the entire measurement period. Thus, the estimated air kerma rates were not affected by rainfall. Air kerma rate due to the gamma-ray energy of natural radiation can be regarded practically as equivalent with the absorbed dose rate in air.

### Activity concentration and the contribution of ^40^K, ^238^U and ^232^Th to air kerma rate

Three measurement points for asphalt-covered surfaces were excluded and the gamma-ray pulse height distributions for the remaining 32 points on soil, grass and beach sand surfaces were unfolded using a 22 × 22 response matrix for the estimations of activity concentrations and the contributions of ^40^K, ^238^U and ^232^Th to air kerma rate [12, 13]. By following the method presented in reference [12, 13], a gamma-ray pulse height distribution obtained by measurements was converted to the energy bin spectrum of incident gamma-rays which is a distribution of gamma-ray flux density to each energy bin. In this method, each channel of a multi-channel analyzer was divided into unequal intervals as energy bins in the gamma-ray energy range from 0 to 3.2 MeV. The gamma-ray energies over 3.2 MeV were not included for evaluation, since the maximum value of the gamma-ray energy from natural radionuclides is 2.615 MeV which is emitted from ^208^Tl. The energy ranges were set up so that the gamma-rays of 1.464 MeV from ^40^K, 1.765 MeV and 2.205 MeV from ^214^Bi (^238^U-series) and 2.615 MeV from ^208^Tl (^232^Th-series) could be stored in each bin, respectively. A 22 × 22 matrix for the 3-in × 3-in NaI(Tl) scintillator for an isotropic field was calculated using the Monte Carlo code, SPHERIX [14, 15]. The gamma-ray flux density and dose rate per unit solid angle are considered almost isotropic in the natural environment [16]. The contribution of cosmic-rays to the gamma-ray pulse height distribution was subtracted using the energy bin number 22 which was stored as the gamma-ray energy range from 3.0 to 3.2 MeV. Moreover, the contribution of ^40^K contamination due to photo-multiplier tube was also subtracted from the gamma-ray pulse height distribution. Clear peaks from ^40^K (bin number 14, energy range: 1.39–1.54 MeV), ^214^Bi (bin numbers 16 and 18; energy range, 1.69–1.84 MeV and 2.10–2.31 MeV) and ^208^Tl (bin number 20; energy range, 2.51–2.72 MeV) were observed in the unfolded spectrum of the gamma-ray pulse height distribution. The activity concentrations of the ^238^U-series, ^232^Th-series, and ^40^K could be estimated by comparing them with the theoretically evaluated gamma-ray flux density spectrum due to these nuclides. In order to evaluate each activity concentration of the natural radionuclides from an energy bin spectrum, it is necessary to calculate the gamma-ray flux densities per unit activity concentrations of the ^238^U-series, ^232^Th-series, and ^40^K. This calculation assumed that a semi-infinite volume source was formed in the ground [13]. The primary and scattered gamma-ray flux density per unit activity concentrations could be calculated using the one-dimensional Monte Carlo gamma transport code, MONARIZA/G2 [17, 18]. A total of a million histories were traced for each natural radionuclide. The nuclear data of gamma-ray energies and disintegration rates used the reported values by Beck [19] and Beck et al. [20] for this Monte Carlo simulation. The activity concentration of each natural radionuclide was evaluated by a successive approximation which used a 3 × 3 matrix determined by Minato [13] to the values of energy bins for ^40^K, ^238^U-series, and ^232^Th-series. Incidentally, the energy resolutions of the photon peak from each natural radionuclide have wide variation for commercially available scintillators. So a diagonal elements fitting (DEF) technique which was developed by Minato [13] was used in this calculation. A new matrix was reconstructed easily and quickly from the standard response matrix using the technique of DEF [13]. The statistical errors for air kerma rate and activity concentrations for ^40^K, ^238^U-series and ^232^Th-series obtained using this software depend on the integral air kerma (nGy) at each measurement point [21], and these were evaluated in this study as 2%, 2%, 6–8% and 4–5%, respectively.

## Results and Discussion

### Shielding factor and dose rate conversion factor

**Fig 2:**
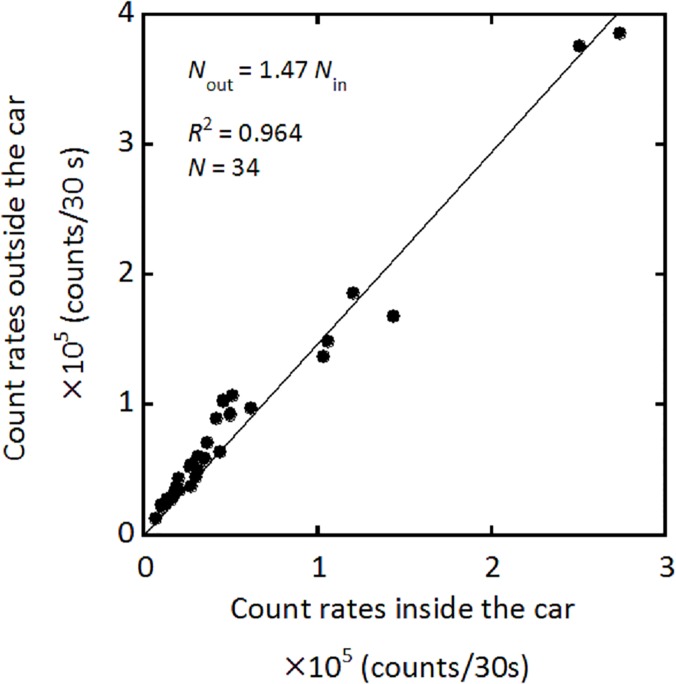
Correlation between count rates outside and inside the car. This regression formula was used as the shielding factor of the car body.

**Fig 3:**
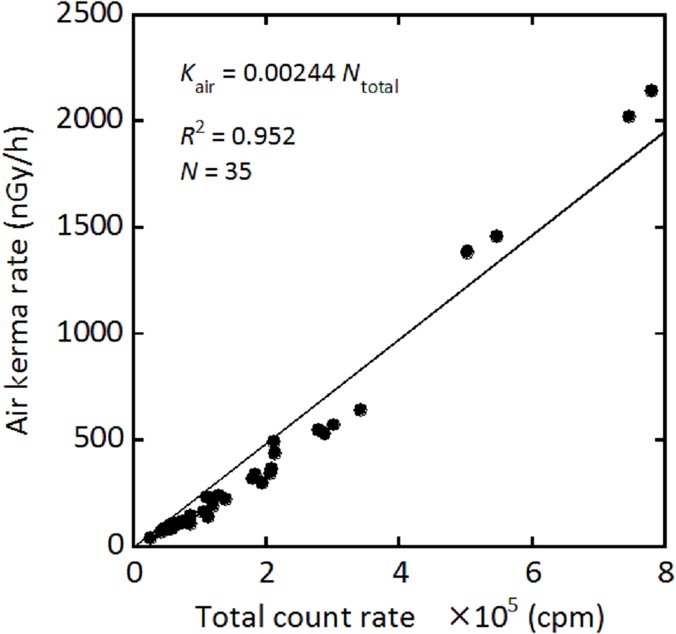
Correlation between air kerma rate which was calculated by software using the response matrix method and total count rate outside the car. This regression formula was used as the dose rate conversion factor.

Fig 2 shows the relationship between the outside and inside count rates, yielding a shielding factor of 1.47. This value was similar to the previously reported value [11]. Fig 3 shows the relationship between air kerma rate (nGy/h) evaluated using the 22 × 22 response matrix method and total count rate (cpm). The conversion factor was evaluated as 0.00244 (nGy/h/cpm). Thus, *K*_out_ the air kerma rate 1 m above the ground surface at each measurement point can be estimated using Eq (1).

$$K_{\text{out}}=2N_{\text{in}}\times 1.47\times 0.00244$$ (1)

In this study, the counts (*N*_in_) inside the car were obtained by the measurements for 30 s. Since the dose rate conversion factor was given as dose rate (nGy/h) for counts per minute (cpm) it is necessary to double *N*_in_ in order to convert into the counts per minute.

### Air kerma rate distribution of Karunagappally Taluk

**Fig 4:**
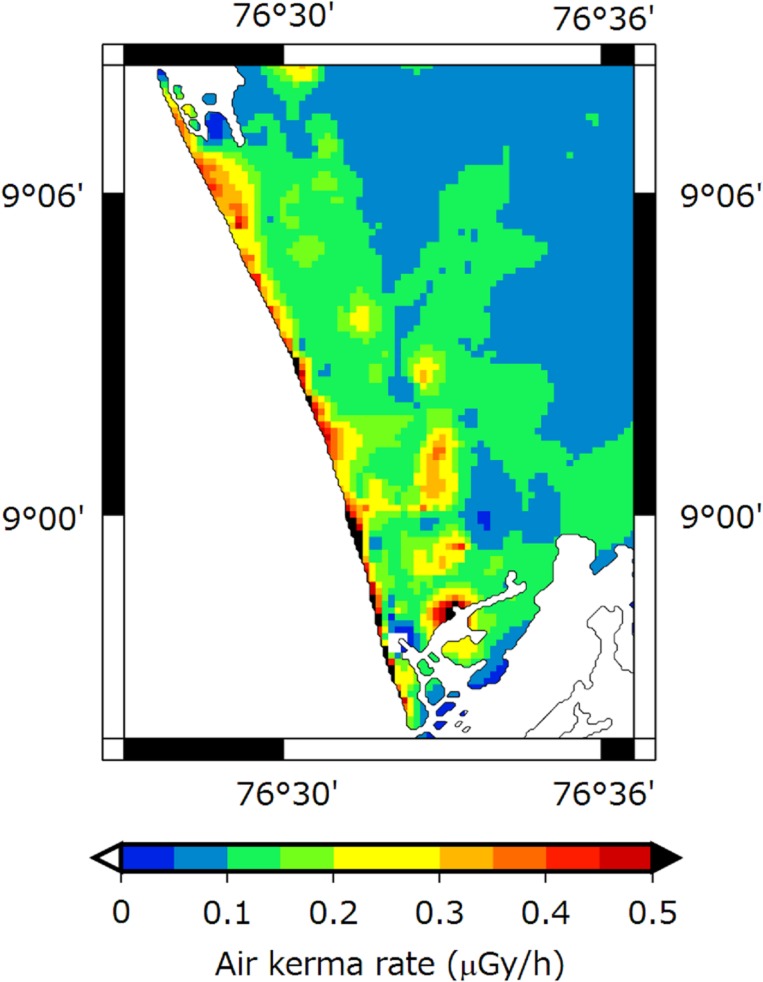
The distribution map of air kerma rate in Karunagappally Taluk. This map was also drawn using GMT [9].

**Fig 5:**
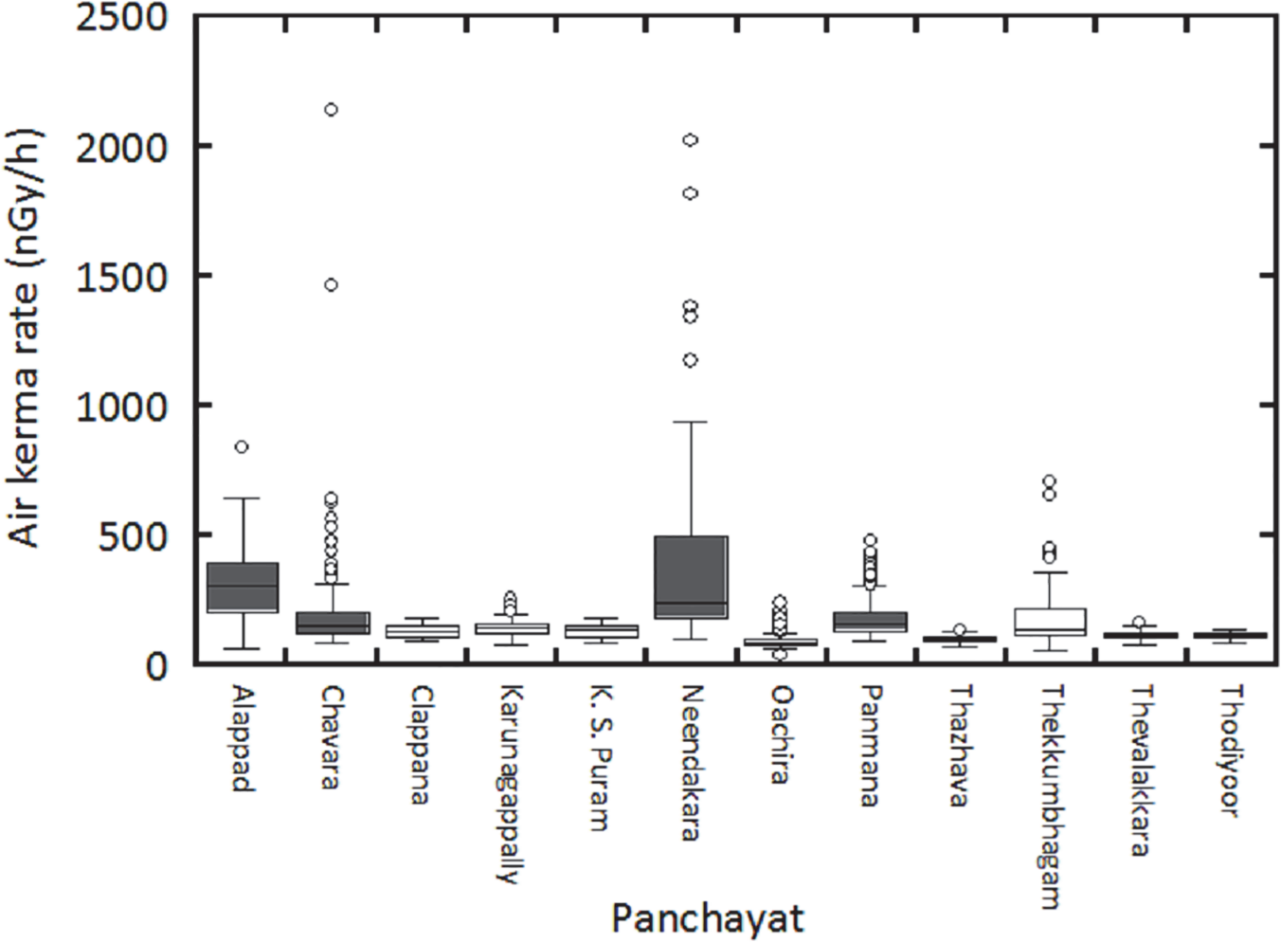
The air kerma rates for each Panchayat as measured by the car-borne survey. Gray shows measurements in high level background radiation areas. White shows measurements for mid-level and low level background radiation areas.

A dose rate distribution map of Karunagappally Taluk is shown in Fig 4. This map was drawn using 2053 data which included the spot measurements. A heterogeneous distribution of air kerma rate was seen. Air kerma rates of over 0.5 μGy/h were found along the coast. However, values of over 0.3 μGy/h were observed in the interior of Karunagappally Taluk. The maximum air kerma rate, 2.1 μGy/h, was observed near the rare earth mining and separation facility in Chavara Panchayat. Moreover, the air kerma rate of 1.9 μGy/h was observed for the coastal area of Neendakara Panchayat. Both these values were observed on sand which likely contained monazite. According to the reports by Nair *et al*. [8] and Christa *et al*. [22], an air kerma rate of about 9 μGy/h was observed for the coastal area of Neendakara Panchayat. Furthermore, Derin, *et al*. [22] observed the maximum value of 28 μGy/h, with an average value of 9.8 μGy/h for the Chavara and Neendakara coastal areas. Since the air kerma rates on the beach sand could not be obtained by the car-borne survey, the maximum values of the present study might be much lower than the value obtained by Derin, *et al*. Thus, the present result suggests that the highest dose rate might be observed in the immediate vicinity of the Lakshadweep Sea. The air kerma rates measured by the car-borne survey for each Panchayat are shown as a box plot in Fig 5. Air kerma rate for high radiation level Panchayats had a large variance. The range of the coefficient of variation for these Panchayats was evaluated to be from 39% to 95%, and these values were relatively higher than those at mid-level and low level Panchayats which ranged from 12% to 32% and from 19% to 58%, respectively. It seems that the higher ^232^Th activity concentration for the coastal areas was a spatially localized distribution.

### Activity concentration

The spot measurement results are shown in Table 1. ^232^Th activity concentration on beach sand was higher than that on soil and grass surfaces, and the range of the activity concentration was estimated to be 0.7–2.3 kBq/kg. According to Derin, *et al*. [23], the highest ^232^Th activity concentration of 41 kBq/kg in beach sand was obtained from coastal areas in Chavara and Neendakara Panchayats and the geometric mean in these areas was estimated to be 14 kBq/kg. In the present study, the contribution of ^232^Th to air kerma rate ranged from 52% to 79%. Especially, the contribution of ^232^Th to air kerma rate was over 70% at measured points with values over 0.34 μGy/h.

**Table 1:** The measured activity concentrations and the contributions of ^40^K, ^238^U and ^232^Th to air kerma rates in Karunagappally Taluk.

Panchayat	Location		Air kerma rate (nGy/h)				Contribution to air kerma rate (%)			Activity concentration (Bq/kg)			Surface
	Latitude (°)	Longitude (°)	^40^K	^238^U	^232^Th	Total	^40^K	^238^U	^232^Th	^40^K	^238^U	^232^Th	Classification (soil, grass, sand)
Alappad	9.0369	76.5087	4	159	471	634	1	25	74	99 ± 2	359 ± 22	716 ± 29	Soil
	9.0524	76.5068	10	25	69	104	10	24	66	240 ± 5	57 ± 3	105 ± 4	Soil
	9.1352	76.4639	28	16	47	91	31	17	52	672 ± 13	36 ± 2	71 ± 3	Soil
Chavara	8.9696	76.5361	1	55	198	254	1	22	78	32 ± 1	124 ± 7	300 ± 12	Soil
	8.9704	76.5450	2	95	334	431	0	22	77	44 ± 1	215 ± 13	507 ± 20	Soil
	8.9877	76.5616	9	24	58	91	10	26	64	214 ± 4	54 ± 3	88 ± 4	Soil
	8.9848	76.5527	4	41	92	137	3	30	67	92 ± 2	93 ± 6	142 ± 6	Grass
	8.9889	76.5314	2	46	166	214	1	21	78	46 ± 1	103 ± 6	253 ± 10	Soil
	8.9698	76.5294	17	163	461	641	3	25	72	407 ± 8	370 ± 22	709 ± 28	Soil
	8.9928	76.5242	40	558	1543	2141	2	26	72	955 ± 19	1269 ± 76	2374 ± 95	Sand
Neendakara	8.9680	76.5316	6	39	113	158	4	25	71	145 ± 3	88 ± 5	172 ± 7	Soil
	8.9634	76.5317	6	98	220	324	2	30	68	151 ± 3	222 ± 13	334 ± 13	Soil
	8.9557	76.5328	25	391	967	1383	2	28	70	594 ± 12	889 ± 53	1488 ± 60	Sand
	8.9557	76.5328	40	521	1460	2021	2	26	72	964 ± 19	1185 ± 71	2247 ± 90	Sand
	8.9534	76.5418	4	76	277	357	1	21	77	102 ± 2	172 ± 10	420 ± 17	Grass
	8.9420	76.5360	5	125	430	560	1	22	77	113 ± 2	283 ± 17	654 ± 26	Sand
Oachira	9.1352	76.5281	6	28	70	104	6	27	67	139 ± 3	64 ± 4	107 ± 4	Soil
	9.1329	76.5338	3	11	28	42	8	26	66	81 ± 2	25 ± 2	42 ± 2	Soil
	9.1167	76.5293	3	18	52	73	4	25	71	66 ± 1	41 ± 2	79 ± 3	Soil
	9.1170	76.5180	2	22	66	90	2	25	73	37 ± 1	51 ± 3	100 ± 4	Soil
	9.1351	76.5088	2	61	215	278	1	22	77	56 ± 1	138 ± 8	327 ± 13	Soil
Panmana	9.0309	76.5501	10	105	322	437	2	24	74	232 ± 5	238 ± 14	496 ± 20	Soil
	9.0019	76.5429	2	69	269	340	1	20	79	53 ± 1	156 ± 9	408 ± 16	Soil
	9.0041	76.5365	4	28	74	106	3	26	70	84 ± 2	64 ± 4	114 ± 5	Soil
	9.0008	76.5254	9	93	244	346	3	27	70	218 ± 4	212 ± 13	375 ± 15	Soil
	9.0162	76.5340	6	37	117	160	4	23	73	135 ± 3	84 ± 5	177 ± 7	Soil
	9.0250	76.5378	1	54	185	240	0	23	77	22 ± 0	123 ± 7	281 ± 11	Soil
Thevalakkara	9.0177	76.5617	5	20	57	82	6	24	70	115 ± 2	45 ± 3	86 ± 3	Soil
	9.0135	76.5881	5	30	93	128	4	24	73	108 ± 2	68 ± 4	142 ± 6	Soil
	9.0106	76.5934	5	33	94	132	4	25	72	112 ± 2	74 ± 4	143 ± 6	Soil
	9.0061	76.5909	2	27	92	121	1	23	76	36 ± 1	62 ± 4	139 ± 6	Soil
	8.9920	76.5746	4	24	72	100	4	24	72	100 ± 2	54 ± 3	110 ± 4	Soil

### External dose estimation

Mean, median, minimum and maximum values of the annual effective dose of each Panchayat are shown in Table 2. It is difficult to make individual dose estimation without considering occupancy factor that depends on the age and sex of the residents [8]. Moreover, it is necessary to consider the shielding factor by the building materials that is used to make the individual dose estimation based on an outdoor dose rate. However, the reported individual dose rate ratios of indoors to outdoors were to be between 0.08 and 1.20 among 197 houses investigated in Kerala state, India [24]. This result suggests that the ratio of 1.20 ± 0.24, which was reported as a typical value for normal background radiation areas in India [25], cannot be used for dose estimation. Individual dose estimation which considers each occupancy factor indoors and outdoors is an issue for future work. Thus, only a conservative external dose estimation was made excluding the occupancy factor and the shielding factor by building materials. The result by this simple approach might be described as an overestimated value. Here, annual effective dose *H* (mSv/y) was estimated using Eq (2):

$$H\;\left(\text{mSv/y}\right)=K_{\text{out}}\times \text{DCF}\times T$$ (2)

where, *K*_out_ is air kerma rate outdoors (nGy/h), DCF is the dose conversion factor (0.7 Sv/Gy) from the air kerma to the external effective dose to adults [5], and *T* is 8760 h (24 h × 365 d). The arithmetic mean of each Panchayat in Karunagappally Taluk ranged from 0.6 mSv/y (Oachira and Thazhava) to 2.3 mSv/y (Neendakara). The highest value (13 mSv/y) was observed in Chavara Panchayat, and a similar value (12 mSv/y) was observed in Neendakara Panchayat. Both these values were observed in beach sand. The annual effective dose less than or equal to 1 mSv/y in Karunagappally Taluk was observed for 69% of all data.

**Table 2:** The estimated annual effective doses of each Panchayat in Karunagappally Taluk.

Panchayat	No. of data	Average (mSv/y)	Maximum (mSv/y)	Minimum (mSv/y)	Median (mSv/y)	Radiation level
Alappad	136	1.9	5.1	0.4	1.9	High
Chavara	214	1.2	13	0.5	0.9	High
Clappana	50	0.8	1.1	0.6	0.8	Middle
Karunagappally	112	0.9	1.6	0.5	0.9	Middle
K. S. Puram	74	0.8	1.1	0.5	0.9	Middle
Neendakara	125	2.3	12	0.6	1.5	High
Oachira	115	0.6	1.5	0.2	0.5	Low
Panmana	273	1.1	2.9	0.6	1	High
Thazhava	138	0.6	0.8	0.5	0.6	Low
Thekkumbhagam	112	1.1	4.3	0.4	0.9	Middle
Thevalakkara	215	0.7	1	0.5	0.7	Low
Thodiyoor	109	0.7	0.9	0.5	0.7	Low

## Conclusions

The car-borne survey with a NaI(Tl) scintillation spectrometer was carried out in Karunagappally Taluk, India, for the purposes of making a dose distribution map and estimating the external dose. The dose rate distribution map showed a heterogeneous distribution of the air kerma rates. Especially, air kerma rates of over 0.5 μGy/h were distributed along coastal areas. The contribution of ^232^Th to air kerma rate at measurement points with values exceeding 0.34 μGy/h was over 70%. The highest annual effective dose of 13 mSv/y was found near the rare earth mining and separation facility in Chavara Panchayat. More than 30% of all the annual effective dose measurements obtained in this study exceeded 1 mSv/y.

## References

[1] Hosoda M, Tokonami S, Tazoe H, Sorimachi A, Monzen S, et al (2013) Activity concentrations of environmental samples collected in Fukushima Prefecture immediately after the Fukushima nuclear accident. Sci Rep 3: 2283 10.1038/srep02283 23887080 PMC3724182

[2] Tokonami S, Hosoda M, Akiba S, Sorimachi A, Kashiwakura I, et al (2012) Thyroid doses for evacuees from the Fukushima nuclear accident. Sci Rep 2: 507 10.1038/srep00507 22792439 PMC3395030

[3] Akahane K, Yonai S, Fukuda S, Miyahara N, Yasuda H, et al (2013) NIRS external dose estimation system for Fukushima residents after the Fukushima Dai-ichi NPP accident. Sci Rep 3: 1670 10.1038/srep01670 23591638 PMC3628369

[4] Ishikawa T (2014) A brief review of dose estimation studies conducted after the Fukushima Daiichi Nuclear Power Plant accident. Radiat Emerg Med 3: 21–27.

[5] United Nations Scientific Committee on the Effects of Atomic Radiation (2014) UNSCEAR 2013 report to the general assembly, with scientific annexes United Nations, New York.

[6] Møller AP, Mousseau TA (2013) The effects of natural variation in background radioactivity on humans, animals and other organisms. Biol Rev 88: 226–254. 10.1111/j.1469-185X.2012.00249.x 23136873

[7] United Nations Scientific Committee on the Effects of Atomic Radiation (2000) UNSCEAR 2000 report to the general assembly with scientific annexes United Nations, New York.

[8] Nair RRK, Rajan B, Akiba S, Jayalekshmi P, Nair MK, et al (2009) Background radiation and cancer incidence in Kerala, India–Karunagappally cohort study. Health Phys 96(1): 55–66. 10.1097/01.HP.0000327646.54923.11 19066487

[9] Wessel P, Smith W (1991) Free software helps map and display data. Eos Trans AGU 72(41): 441–446.

[10] Minato S (1995) Vehicle-borne survey techniques for background radiations. Rep Governmental Industrial Research Institute, Nagoya 44(11): 609–628. (in Japanese)

[11] Minato S, Kawano M (1970) Evaluation of exposure due to terrestrial gamma-radiation by response matrix method, J Nucl Sci Technol 7(8): 401–406.

[12] Minato S (1978) A response matrix of a 3”*φ* × 3” NaI(Tl) scintillator for environmental gamma radiation analysis. Rep Governmental Industrial Research Institute, Nagoya 27(12): 384–397. (in Japanese)

[13] Minato S (2001) Diagonal elements fitting technique to improve response matrixes for environmental gamma ray spectrum unfolding, RADIOISOTOPES 50(10): 463–471.

[14] Matsuda H, Furukawa S, Kaminishi T, Minato S (1982) A new method for evaluating weak leakage gamma-ray dose using a 3”*φ* × 3” NaI(Tl) scintillation spectrometer (I) Principle of background estimation method, Rep Government Industrial Research Institute, Nagoya 31(5): 132–146. (in Japanese)

[15] Minato S (2012) Application of a 60 × 60 response matrix for a NaI(Tl) Scintillator to fallout from the Fukushima reactor accident, Radiat Emerg Med 1(1–2): 108–112.

[16] Minato S (1971) Terrestrial gamma-radiation field in natural environment, J Nucl Sci Technol 8: 342–347.

[17] Minato S (1977) Analysis of the variation of environmental γ radiation during rainfall, Rep Government Industrial Research Institute, Nagoya 26(6): 190–202. (in Japanese)

[18] Minato S (1980) Monte Carlo calculation of gamma radiation field due to precipitation washout of radon daughters from the atmosphere to the ground surface, Jpn J Health Phys 15: 19–24.

[19] Beck HL (1972) The absolute intensities of gamma rays from the decay of ^238^U and ^232^Th, Health and Safety Laboratory Report HASL-262, U.S. Atomic Energy Commission, New York, NY 10014.

[20] Beck HL, DeCampo J, Gogolak C (1972) In-situ Ge(Li) and NaI(T1) gamma-ray spectrometry, Health and Safety Laboratory Report HASL-258, U.S. Atomic Energy Commission, New York, NY 10014.

[21] Matsuda H, Minato S, Pasquale V (2002) Evaluation of accuracy of response matrix method for environmental gamma ray analysis, RADIOISOTOPES 51(1): 42–50. (in Japanese)

[22] Christa EP, Jojo PJ, Vaidyan VK, Anilkumar S, Eappen KP (2012) Radiation dose in the high background radiation area in Kerala, India. Radiat Prot Dosim 148(4): 482–486. 10.1093/rpd/ncr198 21515614

[23] Derin MT, Vijayagopal P, Venkatraman B, Chaubey RC, Gopinathan A (2012) Radionuclides and radiation indices of high background radiation area in Chavara-Neendakara placer deposits (Kerala, India). PLoS ONE 7(11): e50468 10.1371/journal.pone.0050468 23185629 PMC3503973

[24] Chougaonkar MP, Eappen KP, Ramachandran TV, Shetty PG, Mayya YS, et al (2004) Profiles of doses to the population living in the high background radiation areas in Kerala, India. J Environ Radioact 71: 275–297.

[25] Nambi KSV, Bapat VN, David M, Sundaram VK, Sunta CM, et al. (1986) Natural background radiation and population dose distribution in India. Bhabha Atomic Research Centre report. 1–30. Available: http://www.iaea.org/inis/collection/NCLCollectionStore/_Public/20/084/20084715.pdf. Accessed January 26, 2015.

